# Current State and Future Directions of Intranasal Delivery Route for Central Nervous System Disorders: A Scientometric and Visualization Analysis

**DOI:** 10.3389/fphar.2021.717192

**Published:** 2021-07-12

**Authors:** Haiyang Wu, Yan Zhou, Yulin Wang, Linjian Tong, Fanchen Wang, Sirong Song, Lixia Xu, Baolong Liu, Hua Yan, Zhiming Sun

**Affiliations:** ^1^ Clinical College of Neurology, Neurosurgery and Neurorehabilitation, Tianjin Medical University, Tianjin, China; ^2^ Tianjin Key Laboratory of Cerebral Vascular and Neurodegenerative Diseases, Tianjin Neurosurgical Institute, Tianjin Huanhu Hospital, Tianjin, China; ^3^ Department of Ultrasound, Tianjin Huanhu Hospital, Tianjin, China; ^4^ Department of Spine and Spinal Cord, Tianjin Huanhu Hospital, Tianjin, China

**Keywords:** scientometric, intranasal drug delivery, blood-brain barrier, CNS disorders, citespace, VOS viewer

## Abstract

**Background:** The management of various central nervous system (CNS) disorders has been challenging, due to highly compact blood-brain barrier (BBB) impedes the access of most pharmacological agents to the brain. Among multiple strategies proposed to circumvent this challenge, intranasal delivery route has sparked great interest for brain targeting in the past decades. The aim of this study was to apply scientometric method to estimate the current status and future trends of the field from a holistic perspective.

**Methods:** All relevant publications during 1998–2020 were retrieved from the Web of Science Core Collection (SCIE, 1998-present). Two different scientometric software including VOS viewer and CiteSpace, and one online platform were used to conduct co-authorship, co-citation, and co-occurrence analysis of journals, countries, institutes, authors, references and keywords.

**Results:** A total of 2,928 documents, including 2,456 original articles and 472 reviews, were retrieved. Our analysis revealed a significant increasing trend in the total number of scientific publications over the past 2 decades (*R* ^2^ = 0.98). The United States dominated the field, reflecting in the largest amount of publications (971), the highest H-index (99), and extensive international collaboration. Jamia Hamdard contributed to most publications. Frey WH and Illum L were key researchers with the highest number of publications and citations, respectively. The *International Journal of Pharmaceutics* was the most influential academic journal, and Pharmacology/Pharmacy and Neurosciences/Neurology were the hottest research categories in this field. Based on keywords occurrence analysis, four main topics were identified, and the current research focus of this field has shifted from cluster 4 (pathways and mechanisms of intranasal delivery) to cluster 2 (the study of nasal drug delivery systems), especially the nanostructured and nano-sized carrier systems. Keywords burst detection revealed that the research focus on oxidative stress, drug delivery, neuroinflammation, nanostructured lipid carrier, and formulation deserves our continued attention.

**Conclusion:** To the authors’ knowledge, this is the first scientometric analysis regarding intranasal delivery research. This study has demonstrated a comprehensive knowledge map, development landscape and future directions of intranasal delivery research, which provides a practical and valuable reference for scholars and policymakers in this field.

## Introduction

Central nervous system (CNS) disorders, including neurodegenerative, neoplastic, and neuropsychiatric disorders are becoming increasingly prevalent around the world due to rapidly aging populations (Hebert et al., 2013). Treatment of various CNS disorders has always been a challenging task, despite the rapid development of multiple novel treatment strategies and drug approaches in recent years. Of these, development of efficient brain targeted drug delivery system is crucial for successful therapy of CNS diseases. However, the presence of the complex blood brain barrier (BBB) is one of the major obstacles that limits therapeutics entry to the CNS region (Jafari et al., 2019). The BBB, comprises brain microvascular endothelial cells, pericytes, and astrocytic end-feet, acts as a selective physical barrier that prevents approximately 98% of small molecular weight agents and nearly 100% of macromolecules, from crossing from the blood stream into the brain tissues (Pardridge, 2005; Jafari et al., 2019; Wong et al., 2019).

Consequently, numerous studies have proposed promising strategies to improve the delivery of therapeutic agents to the brain by circumventing or overcoming the BBB during the last decades. These strategies include intracerebroventricular injections (Lopachev et al., 2019), convection enhanced delivery (Zhan and Wang, 2018), inhibition of efflux transporters (Sanchez-Covarrubias et al., 2014), chemical modification (Al-Ahmady, 2018), and the application of hyperosmotic agents (Patel et al., 2009) or focused ultrasound (Dauba et al., 2020; Lin et al., 2020; Wu et al., 2021a), and so on. However, the limitation of these administration routes is marred by safety concerns, invasive, and erratic efficacy. For example, despite the approach of osmotic disruption, works by shrinking endothelial cells and opening tight junctions, can improve the delivery efficiency of drugs to large brain regions, non-selective BBB opening by mannitol can also result in various side-effects such as epilepsy, brain edema, and hypotension (Patel et al., 2009; Al-Ahmady, 2018). Other methods like convection enhanced delivery, remain in experimental stages and several critical issues need to be resolved, including determining optimal infusion volume and rate (Zhan and Wang, 2018).

Furthermore, besides the methods mentioned above, the intranasal route has emerged as a promising approach to transport therapeutic biomolecules directly from nose-to-brain, bypassing the BBB (Alam et al., 2010; Freiherr et al., 2013; Antunes-Viegas et al., 2016). It represents a non-invasive route of brain drug delivery compared with intravenous administration and direct intracerebroventricular injection. In addition, drugs administered into the nasal cavity can also overcome the limitations of the oral administration by avoiding the usual enzymatic degradation and hepatic first-pass effect, resulting in the enhancement of drug bioavailability and the rapid onset of pharmacological action (Singh et al., 2013; Fortuna et al., 2014; Erdő et al., 2018; Micheli et al., 2020). Taken together, this route has shown several advantages of non-invasiveness, higher bioavailability, rapid onset of action and easiness of practical application. Although the exact mechanisms by which the molecules are transported directly from the nasal cavity to the CNS remain unclear, the involvement of cerebrospinal fluid, neuronal pathways, and nasal lymphatics has been evidenced through plenty of studies (Alam et al., 2010; Crowe et al., 2018; Gänger and Schindowski, 2018). Given the importance of intranasal delivery technique in CNS diseases, it has gained significant interest for brain targeting and numerous exciting studies have been performed in recent years (Gonçalves et al., 2019; Pires et al., 2021). In the meantime, some scientific researchers have focused on reviewing relevant published literature to summarize the characteristics and status of intranasal delivery research. However, almost all of the reviews were about a branch or a specific subfield of intranasal delivery research, and little attention was paid to estimate the current status and future trends of the field from a holistic perspective (Alam et al., 2010; Ali et al., 2010; Gomez et al., 2012; Freiherr et al., 2013).

Scientometric analysis has emerged as a powerful tool in reflecting changes of a discipline during its development. Unlike systematic reviews, scientometrics refers to the quantitative analysis of literature information using mathematical and statistical methods, and has been often used with the intention of identifying the overall knowledge framework, evaluating current status and predicting future directions within a given field (Chen et al., 2014a; De-Castilhos-Ghisi et al., 2020). Recently, several bibliometric visualization tools, like HistCite (Garfield et al., 2006), VOS viewer (van-Eck and Waltman, 2010), Citespace (Synnestvedt et al., 2005), and RBibliometrix (Ke et al., 2020), have been widely used in various disciplines of scientific research, including material science (Caixeta et al., 2020), food industry (Blázquez-Ruiz et al., 2016), ecology (De-Castilhos-Ghisi et al., 2020), and biomedicine (Chen et al., 2010; Deng et al., 2020; Yeung et al., 2021). Nevertheless, as far as we know, there are no prior reports on scientometric studies covering researches on intranasal delivery, regardless of a large body of published literature on this scientific area that can provide important references to promote further research in this field.

In view of this, we conducted a scientometric analysis of studies on intranasal delivery research published from 1998 to 2020. The aims of this study were to identify the major players and their cooperation networks, including countries, academic groups, and individuals; to analyze research status and hotspots, especially the key study findings in this field; to summarize the main research themes and clusters, discuss the potentially valuable research directions, including drug delivery systems (Ali et al., 2010; Battaglia et al., 2018; Pires et al., 2020) and the broad prospect of clinical application.

## Materials and Methods

### Data Source and Search Strategy

Scientometric analysis was conducted using the Science Citation Index Expanded (SCIE) database of Web of Science Core Collection (WoSCC, Clarivate Analytics, 1998-present, http://lib.tmu.edu.cn/), which contains more than 12,000 of the highest impact international journals, and is considered as the most prominent bibliographic databases of peer-reviewed scientific publications on many research topics (Merigó and Yang, 2017; Li et al., 2021; Zhang et al., 2021). The search terms and retrieval strategies were developed as follows: # 1, topic: (intranasal administra* OR intranasal deliver* OR nasal administra* OR nasal deliver* OR “nose to brain”); # 2, topic: (brain OR “central nervous system” OR CNS); # 3, “# 1” AND “# 2.” Wildcard “*” indicates any group of characters or no character, for instance, “deliver*” would also return “delivery” or “deliveries” (Willem et al., 2017). The searched time span was from 1998 to 2020, the language type was limited to English, and the document type was set to articles or reviews. In addition, database search was conducted on a single day, April 25, 2021, so as to avoid the possible bias came from the significant fluctuations in the numbers of studies, as well as citations.

### Data Extraction and Collection

**FIGURE 1:**
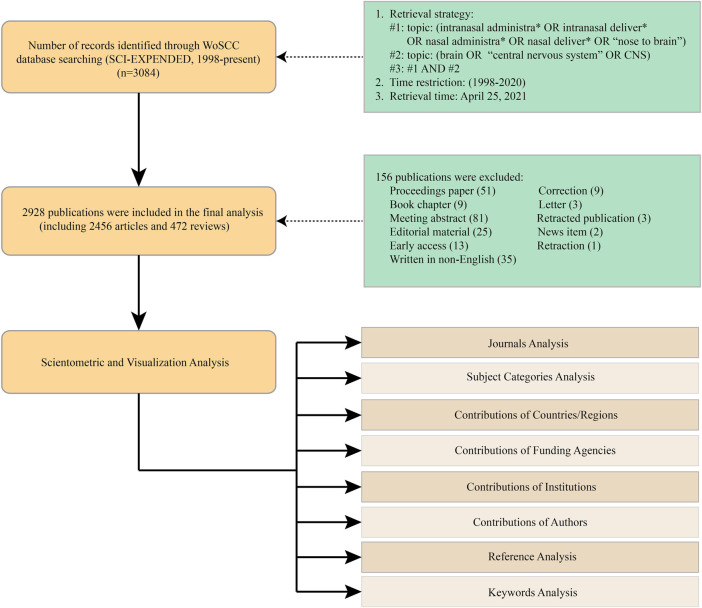
The steps of literature identification and screening process.

Following the retrieval strategies stipulated above, a total of 2,928 studies were initially obtained. The bibliographic records of these studies, including titles, authors, abstracts, and cited references, were first exported into CiteSpace software in the form of plain text to remove any duplicates, and then sent to scientometric software, Microsoft Excel 2019 and statistical software for subsequent analysis. In WoSCC, the online “Analyze Results” function was applied to preliminarily analyze general information about distribution of annual publications, journals, countries, organizations, authors, funding agencies and research areas. And other information, such as the sum of times cited, average citations per item (ACI) and H-index was also acquired by using the “Create Citation Report” function. Journal impact factor (IF) was retrieved from the Journal Citation Reports (JCR) published in 2019. Figure 1 describes the steps of literature search and selection process.

### Data Analysis

#### Statistical Analyses

This study used Microsoft Excel 2019 (Microsoft Corporation, Redmond, Washington, United States) and SPSS 21 (IBM SPSS Statistics 21, Inc., Chicago, IL, United States) to classify, descriptively analyze, and statistically evaluate the data extracted from the literature. The growth rate of publications with time was calculated by raising the rate of the quantity of scientific output in 2020 over the quantity of scientific output in 1998 to the power of 1/23, and the specific calculation formula was shown as follows: Growth rate = [(quantity of publications in the last year ÷ quantity of publications in the first year)^1/(last year—first year)^ - 1] × 100 (Guo et al., 2020). Pearson’s correlation coefficient was used to test a possible correlation between the quantity of scientific output and the geographic/economic features of countries. And a significant correlation existed when *p*-value < 0.05. Latest Gross Domestic Product (GDP) and demographic data (2019) were obtained from the World Bank official website and the website of the Central People’s Government of the People’s Republic of China. Also, GraphPad Prism 8, and R software (v3.6.3.) were used for mapping and data visualization.

#### Scientometric and Visualization Analysis

CiteSpace V (Version 5.7. R5) and VOS viewer (Version 1.6.16), were used to perform scientometric analysis and network visualization. CiteSpace is a free Java-based application designed by Chen (Synnestvedt et al., 2005). In this study, CiteSpace was used to conduct research cooperation relationships of institutions, co-occurring network of subject categories, the dual-map overlay of journals, reference and keyword bursts. The indicator of betweenness centrality of a node can measure the importance of each node in the network, and the node with a higher centrality is commonly considered as a key point or pivotal turning point in the field (Wu et al., 2021a; Wu et al., 2021b). For additional detailed information about CiteSpace software, a manual is available at http://cluster.ischool.drexel.edu/∼cchen/citespace/CiteSpaceManual.pdf.

Then, we use VOS viewer software, another bibliometric software and visualization tool developed by Professor Eck and Waltman, to create co-authorship, co-citation, and co-occurrence networks in the field of intranasal delivery research (van-Eck and Waltman, 2010). Specifically, country/author co-authorship analysis, journal/author co-citation analysis and keyword co-occurrence analysis were performed and three visualization maps, including the network visualization map, the overlay visualization map, and the density visualization map were constructed in this study. Generally, the circle labels in the visualization map represent different parameters such as authors, countries, journals and keywords. The node size is determined by the number of publications, citations or occurrences. The links between the nodes represent the correlation between parameters, and the strength of links is assessed quantitatively by the indicator of total link strength (TLS) (Li et al., 2021; Zhang et al., 2021). Detailed explanations of these visualization maps are provided in the figure legends and also available at https://www.vosviewer.com/documentation/Manual_VOSviewer_1.6.16.pdf. The parameter settings of VOS viewer were as follows: type of analysis (choose one at a time), counting method (fractional counting), thresholds of items (depending on particular situations and detailed values were provided in the **Results** section), VOS viewer thesaurus file (merge different variants of country/organization names or keywords).

In addition to the software mentioned above, an online bibliometric analysis platform (https://bibliometric.com/) was also used to conduct research cooperation relationships between countries.

### Research Ethics

Ethical approval was not required for this study, as all data were downloaded from public databases and did not involve any human or animal participants.

## Results and Discussion

### Growth Trend of Annual Publication and Citation Quantity

By applying the aforementioned search strategy and selection process, a total of 2,928 related documents including 2,456 original articles and 472 reviews were collected in a period from 1998 to 2020. The cumulative total citations for all publications were 98,904 times (76,372 times without self-citations), an average of approximately 33.78 per document, and H-index of all the literature was measured as 124.

**FIGURE 2:**
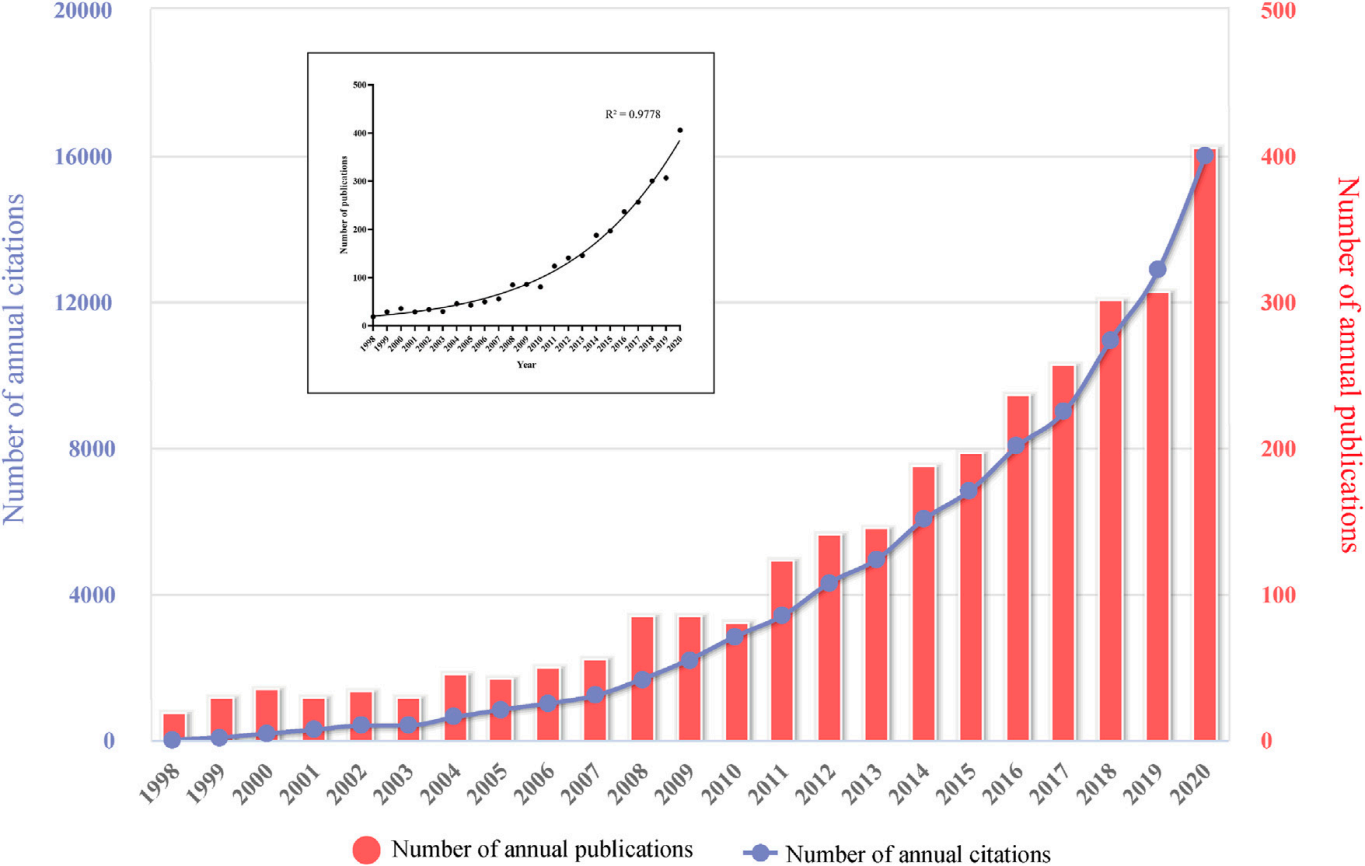
Distribution of the annual published documents and citations on intranasal delivery research from 1998 to 2020.

The changing trend of annual publications and citations of intranasal delivery research is displayed in Figure 2. Our analysis revealed a significant increasing trend in the total number of scientific publications over the past 23  years (*R* ^2^ = 0.98). From 1998 to 2020, the average growth rate of scientific publications on intranasal delivery research was 14.49%. The number of publications has increased from 19 in 1998 to 406 in 2020, and almost 51.5% of them were published in the last five years. When it comes to the citation number, it could be seen from the chart that the increasing trend for the annual citation number showed a similar trend as the annual publication number. Upon plotting publication vs. citation (Supplementary Figure S1), a significant linear correlation could be deduced with a satisfactory correlation coefficient (*r* = 0.995) and explicability (*R* ^2^ = 0.99). Accordingly, the majority of variations in the citation rate can be explained by the publication rate.

From the above results, it is clear that, with the deepening of research in recent years, there has been an explosion of interest in the role of intranasal administration as a non-invasive drug delivery technique for CNS diseases, either from the annual volume of publication or from the annual citation perspective.

### Overall Knowledge Framework of Global Publications

#### County/Region Analysis

**FIGURE 3:**
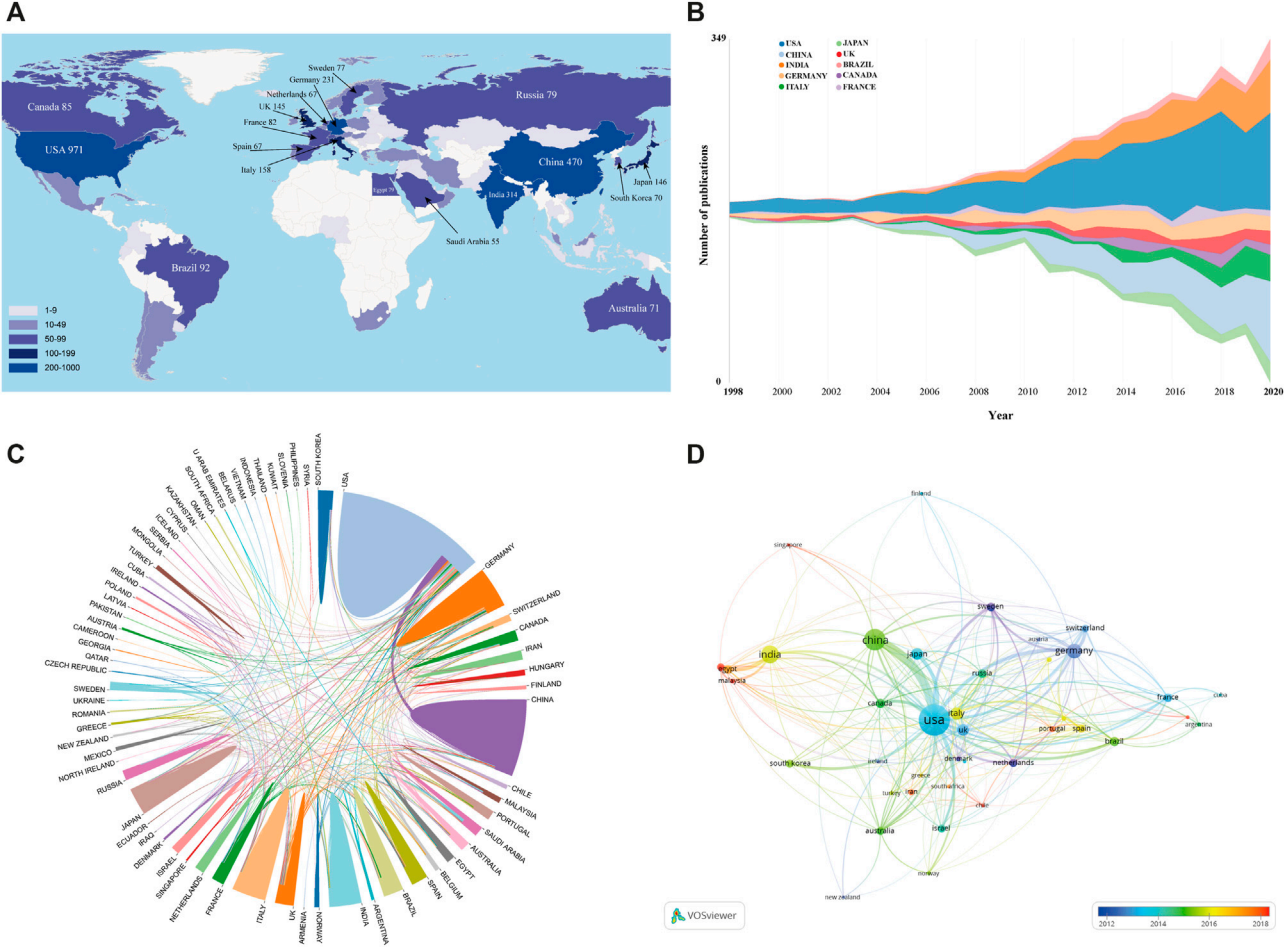
**(A)** Geographical distribution based on the total number of scientific publications about intranasal delivery research. **(B)** The annual publications of the top 10 countries from 1998 to 2020. **(C)** The international cooperation among relevant countries/regions. **(D)** The country co-authorship overlay visualization map using VOS viewer. Each node represents an individual country, and the node size is proportional to the number of publications. Line thickness between nodes indicates link strength of a collaboration relationship (weighted by a quantitative evaluation indicator of TLS).

Of these 2,928 publications, researchers from 76 countries have so far contributed to this field. These countries include developing and developed economies. Among them, the United States published the maximum number of research articles pertaining to intranasal delivery research (*n* = 971) followed by China (*n* = 470) and India (*n* = 314) (Figure 3A and Table 1). Apart from that, when adjusted by populations and GDP, Israel was both the top one with 7.62 papers per million populations and 176.92 papers per trillion GDP. Supplementary Table S1 shows a very highly significant positive correlation between the number of publications related to intranasal delivery research and GDP. The predictive equation had a good fit with an *R* ^2^ = 0.904. While the factor of total population size had little correlation with the number of publications and the explicability of the equation was very low (*R* ^2^ = 0.236). Therefore, it can be concluded that much of the variation in the number of publications among different countries could be explained by economic factors. After all, we all know that the development of natural science cannot be separated from financial support.

**TABLE 1:** Top 20 most productive countries in the research field of intranasal administration.

Ranking	Countries	Output, n (%)	Number of papers per trillion GDP	Number of papers per million people	H-index	ACI
1	United States	971 (33.16)	45.31	2.96	99	44.88
2	China	470 (16.05)	30.58	0.33	57	26.48
3	India	314 (10.72)	109.41	0.23	50	28.55
4	Germany	231 (7.89)	59.84	2.78	57	56.50
5	Italy	158 (5.40)	79.00	2.62	36	25.75
6	Japan	146 (4.99)	28.74	1.16	35	24.11
7	United Kingdom	145 (4.95)	51.24	2.17	41	47.54
8	Brazil	92 (3.14)	50.00	0.44	25	22.09
9	Canada	85 (2.90)	48.85	2.26	28	29.27
10	France	82 (2.80)	30.15	1.22	28	31.06
11	Egypt	79 (2.70)	263.33	0.79	23	20.73
12	Russia	79 (2.70)	46.47	0.55	13	15.20
13	Sweden	77 (2.63)	145.28	7.48	32	38.29
14	Australia	71 (2.42)	50.71	2.80	27	41.08
15	South Korea	70 (2.39)	42.42	1.35	22	21.49
16	Israel	69 (2.36)	176.92	7.62	28	42.93
17	Netherlands	67 (2.29)	73.63	3.87	30	39.34
18	Spain	67 (2.29)	48.20	1.42	21	20.01
19	Switzerland	62 (2.12)	88.57	7.23	30	102.45
20	Saudi Arabia	55 (1.88)	69.62	1.60	18	15.29

Ranking: according to the number of total papers. GDP: Gross Domestic Product. ACI: Average Citations per Item. Publications from Taiwan, Hong Kong, and Macau were assigned to China, and those from England, Northern Ireland, Scotland, and Wales were reclassified to the United Kingdom.

In terms of the H-index, the United States still ranked first with 99, followed by China and Germany, which all had an H index above 50 (Table 1). H-index, defined as the number of papers with citation number ≥ h, is an important indicator to simultaneously characterize the quality and quantity of scientific output (Hirsch, 2005; Merigó and Yang, 2017). Thus, it is one of the dominant metrics for quantifying the productivity and impact of an author, a country or an institution (De-Castilhos-Ghisi et al., 2020; Wu et al., 2021a). Our study revealed that the United States was the leading country both in the total number of publications and H-index. Therefore, whether from a research quality or from quantity point of view, the United States was dominated in this research field. Moreover, it is worth noting that although there were few papers published in some countries, there were many average citations per item (ACI) obtained. For example, Switzerland occupied the top ranked position in the aspect of average citations per item (ACI) (*n* = 102.45 times) and much higher than other countries. This could have been relevant to the publication of some highly influential studies. Unsurprisingly, one study from Switzerland titled “Oxytocin increases trust in humans” has received more than 2000 citations, which is ranked first among the top 10 high-cited articles in this domain (Kosfeld et al., 2005).

Figure 3B summaries the annual publications of the top 10 countries from 1998 to 2020. It can be seen that the United States was always the most prolific contributing country over the study period, while China and India started rapid development in the 2010s. Figure 3C displays international cooperation among relevant countries/regions. It is comforting to note that active collaboration was observed between developed and developing countries. For instance, authors from the United States collaborated most closely with authors from China, Germany, and Japan. China, Germany, Canada and United Kingdom demonstrated active cooperation as well. Figure 3D illustrates the country co-authorship overlay visualization map using VOS viewer software. Only countries/regions with more than 10 papers were included. Of the 40 countries/regions that met this threshold, the top 3 with the largest TLS were the United States, China and Germany, which further proved the dominant position of these countries in this field. Additionally, as also can be seen from this map, a node's color indicates the average appearing year (AAY) of each country according to the color gradient shown in the lower right corner. We can find that the countries of Sweden (AAY = 2009.03), Netherlands (AAY = 2011.35), Denmark (AAY = 2011.54) are given dark blue color, which indicates that most participants of these countries entered in the early stage of intranasal delivery research. While Mexico, Chile and Malaysia were with AAY of 2018.87, 2018.35 and 2018.31, respectively, which implies that they were relatively new entrants in this field.

#### Funding Agency Analysis

**FIGURE 4:**
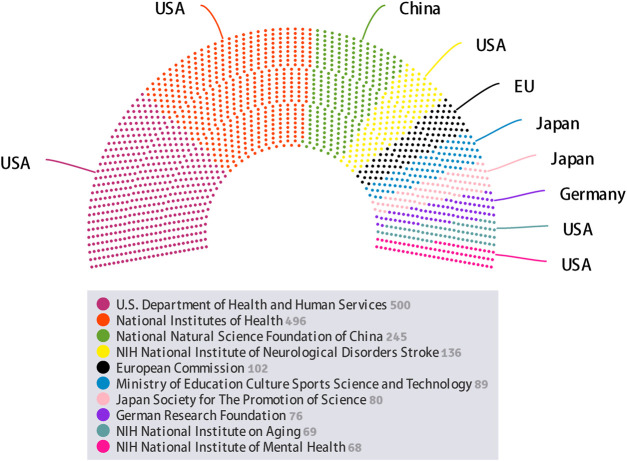
The top 10 related funding agencies according to the number of funded projects.

Undoubtedly, development of intranasal delivery research requires significant financial investment. In view of this, we have briefly summarized the funding information in this area. Figure 4 illustrates the top 10 related funding agencies for the support of intranasal delivery research. From the distribution of funding agencies, half of them were from the United States, two from Japan, and the remaining three agencies were from China, European Union, and Germany. Specifically, the fund project of U.S. Department of Health and Human Services (HHS) has sponsored the largest number of research (of 500 studies funded). National Institutes of Health (NIH) and National Natural Science Foundation of China (NSFC) rank in the second and the third place, with 496 and 245 studies, respectively. The results above illustrate that developed countries especially the United States have taken a dominant position in the field of intranasal delivery research as they are investing substantial capital into developments in this technology. In general, adequate funding represents a necessary but not sufficient requirement for engaging and retaining new talent, research team to devote more work to a certain field (Wu et al., 2021b).

#### Institution Analysis

**FIGURE 5:**
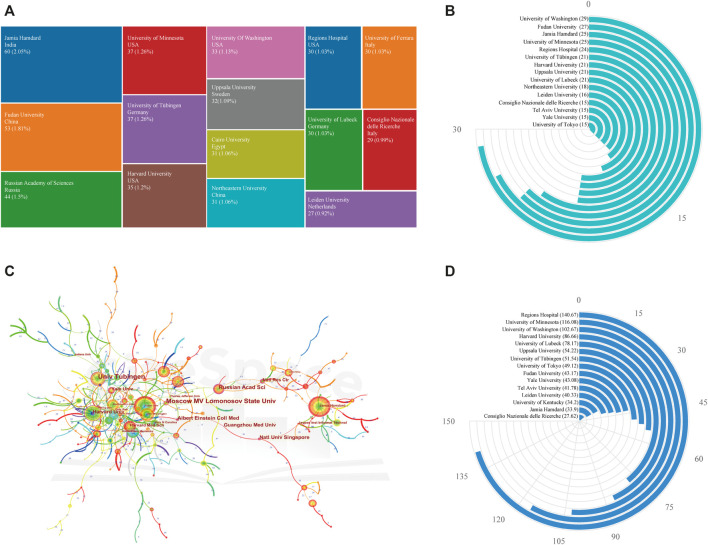
**(A)** The top 15 most prolific institutions and the quantity of publications in each institution. **(B)** The top 15 institutions with the highest value of H-index. **(C)** The network visualization map for institutions’ cooperation generated by CiteSpace. Each node represents an individual institution, and the node size represents the number of publications, and the larger the node, the greater the number of publications. The size of the label is proportional to the centrality. The line between nodes represents the collaboration between institutions and the value of links strength was also added between lines. **(D)** The top 15 institutions with the highest value of ACI.

As for institutions analysis, a total of 2,759 institutions have made contributions to the published research in the field. The top 15 most prolific institutions and the quantity of publications in each institution are presented in Figure 5A. As can be seen, within the list of top 15 institutions, there were six institutions from Western Europe, four American institutions, three Asian institutions, one Russian as well as one Egyptian institution. Jamia Hamdard was the largest contributor in terms of numbers of publications (N = 60, 2.05%), followed by Fudan University and Russian Academy of Sciences, with 53 and 44 publications, respectively. Beyond that, academic institutions should not only value the number of publications, but more attention should be paid to the quality of the publications. As shown in Figure 5B, University of Washington had the highest value of H-index (29), followed by Fudan University (27), University of Minnesota (25), and Jamia Hamdard (25). In terms of ACI (Figure 5D), the top three institutions with largest ACI were Regions Hospital (140.67 times), University of Minnesota (116.08 times), and University of Washington (102.67 times). It is worth noting that there is no institute with an absolute leading position at about the same time in publication outputs, H-index, and ACI.

The network visualization map for institutions’ cooperation was generated by CiteSpace (Figure 5C). University of Tübingen had the highest centrality, with a central value of 0.16, followed by Moscow M. V. Lomonosov State University (0.15) and Russian Academy of Sciences (0.13). Besides, as can be seen from this map, there is a certain amount of cooperation and exchange between different research institutions around the world, and the cooperation between institutions in Europe and America was relatively close. But the cooperation among other institutions was not very close, mostly restricted either to a country or a particular region.

#### Author Analysis

**FIGURE 6:**
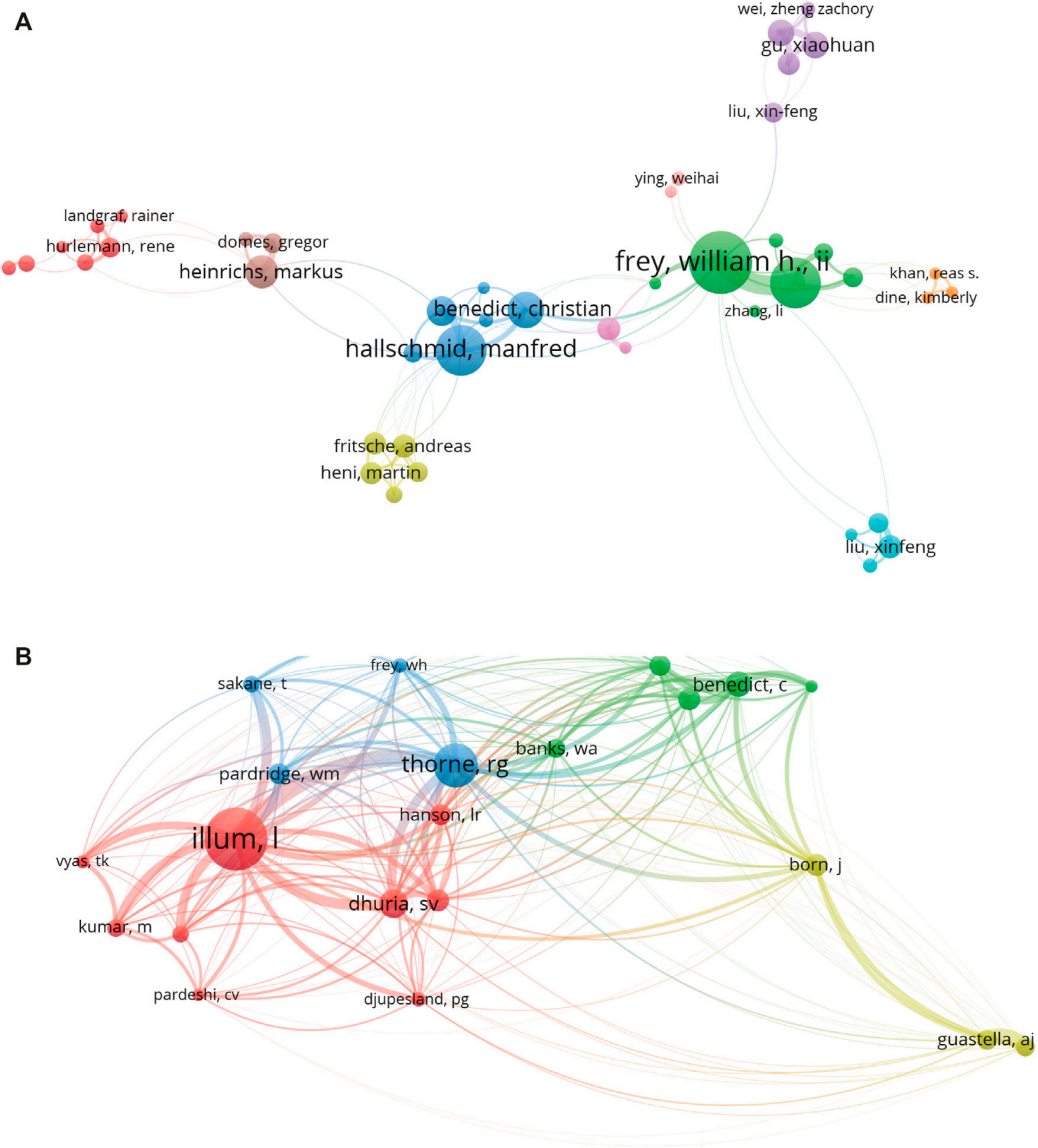
**(A)** Visualization map of the 288 identified authors by co-authorship analysis based on VOS viewer software. Explanatory figure legend is the same as for Figure 3D. **(B)** Mapping on the co-citation analysis among 21 identified authors devoted to intranasal delivery research. Unlike co-authorship network map, the node size is proportional to citation frequency. A line between two nodes indicates that both were cited by one author.

Number of scientific publications made by authors, to some extent, represents the contribution and activity of authors in this area. The top 10 most productive authors based on the number of publications contributed 355 papers (12.12%) on intranasal delivery research (Table 2). Frey WH (Thorne and Frey, 2001; Reger et al., 2005) from Health Partners Neuroscience Research contributed the most articles (51), followed by Ali J and Baboota S (Alam et al., 2010; Alam et al., 2012; Fazil et al., 2012) from Jamia Hamdard University, with 48 and 44 publications, respectively. Most authors only contributed one or two papers, implying that only a few researchers have made a substantial contribution to this field. From the author co-authorship network map (Figure 6A), only authors with a minimum of five publications were included. There were 288 nodes in the network map. Ali J, Baboota S, and Frey WH were located at a central position of the co-authorship map. Nevertheless, it is obvious that cooperation among authors is not very common, only with 10 main clusters and mostly linking with 3–5 authors. Therefore, more attention should be paid to improve the communication among research teams. The above-mentioned findings also enable scholars to identify active research communities in this area and seek out potential academic collaboration.

**TABLE 2:** The top 10 authors with the largest number of publications and citations in intranasal administration research.

Ranking	Author	Output, n (%)	H-index	Country/Region	Co-cited author	Citations	TLS
1	Frey W. H.	51 (1.74)	31	United States	Illum L.	1,174	775.00
2	Ali J.	48 (1.64)	24	India	Thorne R. G.	810	650.34
3	Baboota S.	44 (1.50)	23	India	Dhuria S. V.	499	428.70
4	Hallschmid M.	40 (1.37)	27	Germany	Benedict C.	448	376.53
5	Born J.	37 (1.26)	23	Germany	Lochhead J. J.	394	333.71
6	Hanson L. R.	34 (1.16)	20	United States	Born J.	386	337.25
7	Jiang X. G.	28 (0.96)	20	China	Craft S.	378	297.76
8	Chen J.	26 (0.89)	16	China	Reger M. A.	371	324.97
9	Benedict C.	25 (0.85)	20	Sweden	Hanson L. R.	363	304.36
10	Liu X. F.	22 (0.75)	20	China	Pardridge W. M.	358	253.52

In addition, we also use VOS viewer software to perform author co-citation analysis (Figure 6B). Co-citation analysis refers to the relationship of items built based on the number of times they are cited together by a third citing item (Deng et al., 2020). Authors were included if the minimum number of citations was over 200 times. There were 21 authors that met the criteria. The top five authors with the greatest TLS were as follows: Illum L (Illum, 2000; Illum, 2003), Thorne RG (Thorne and Frey, 2001; Thorne et al., 2004), Dhuria SV (Dhuria et al., 2009; Dhuria et al., 2010), Benedict C (Benedict et al., 2004; Benedict et al., 2008), and Born J (Benedict et al., 2004; Hallschmid et al., 2004). The results indicated that these authors and their research teams have the highest research strength and influence in this field, and they are more likely to publish significant findings that are favorable to intranasal delivery research than others. Take Illum L as an example, he has authored 16 articles and reviews in this field which have been cited over 3,006 times with the ACI of 187.88 times calculated from the data of WoSCC. Of these, two of these papers had been cited over 500 times, which mainly discussed the problems associated with nasal drug delivery and examples of studies in man (Illum, 2000; Illum, 2003). Moreover, it is interesting to note that the three most productive authors did not show up in the top three most cited authors. This illustrates that number of articles may not reflect the academic influence of an author, which is a common problem in developing countries, such as China. Some scholars have put forward that although the number of Chinese papers published in international journals has greatly increased, high-quality research papers published in top grade journals are not very frequent (Qu et al., 2008; Wang et al., 2019). It is worth mentioning that this problem has gained the attention of policy-makers in China and academics are encouraged to improve research quality, rather than the quantity (Wu et al., 2021b).

#### Journal Analysis

An academic journal distribution analysis is helpful for scholars to determine the dominant journals in a certain field, and to select the most appropriate scientific journals for publication of their research results (Li et al., 2021; Wu et al., 2021a). Thus, we have listed the 10 journals with the most publications related to intranasal administration research in Table 3. These 10 prolific journals totally published 449 papers, accounting for 15.33% of all 2,928 publications. *International Journal of Pharmaceutics* published the most articles (*n* = 83), *Plos One* ranked the second, with 50 publications, followed by *Drug Delivery* (*n* = 47), *Pharmaceutics* (*n* = 46), and *Journal of Controlled Release* (*n* = 44). It is not difficult to find that research outputs are mainly published in these journals focused on Pharmacology and Pharmacy. Scholars in the field are perhaps more oriented to publish the latest research findings in these journals in future work. In terms of IF, *Journal of Controlled Release* has the largest impact factor of 7.727, followed by *Drug Delivery* (4.902) and *International Journal of Pharmaceutics* (4.845). According to the corresponding JCR categories published in 2019, seven and three journals are categorized in Q1 and Q2, respectively. Moreover, all the top 10 journals were hosted by Western European and North American countries especially Netherlands, England, and the United States, which could be an important factor for Euro-American countries dominating this field.

**TABLE 3:** Top 10 journals with most publications related to intranasal administration research.

Ranking	Journal title	Research topics	Output, n (%)	IF (2019)	Quartile in category (2019)
1	*International Journal of Pharmaceutics*	Pharmacology & Pharmacy	83 (2.83)	4.845	Q1
2	*Plos One*	Science & Technology	50 (1.71)	2.74	Q2
3	*Drug Delivery*	Pharmacology & Pharmacy	47 (1.61)	4.902	Q1
4	*Pharmaceutics*	Pharmacology & Pharmacy	46 (1.57)	4.421	Q1
5	*Journal of Controlled Release*	Chemistry	44 (1.50)	7.727	Q1
		Pharmacology & Pharmacy			
6	*Psychoneuroendocrinology*	Endocrinology & Metabolism/Psychiatry	38 (1.30)	4.732	Q1
		Neurosciences & Neurology			
7	*Journal of Pharmaceutical Sciences*	Pharmacology & Pharmacy	37 (1.26)	2.997	Q2
		Chemistry			
8	*Scientific Reports*	Science & Technology	36 (1.23)	3.998	Q1
9	*Expert Opinion on Drug Delivery*	Pharmacology & Pharmacy	34 (1.16)	4.838	Q1
10	*Journal of Drug Targeting*	Pharmacology & Pharmacy	34 (1.16)	3.38	Q2

**FIGURE 7:**
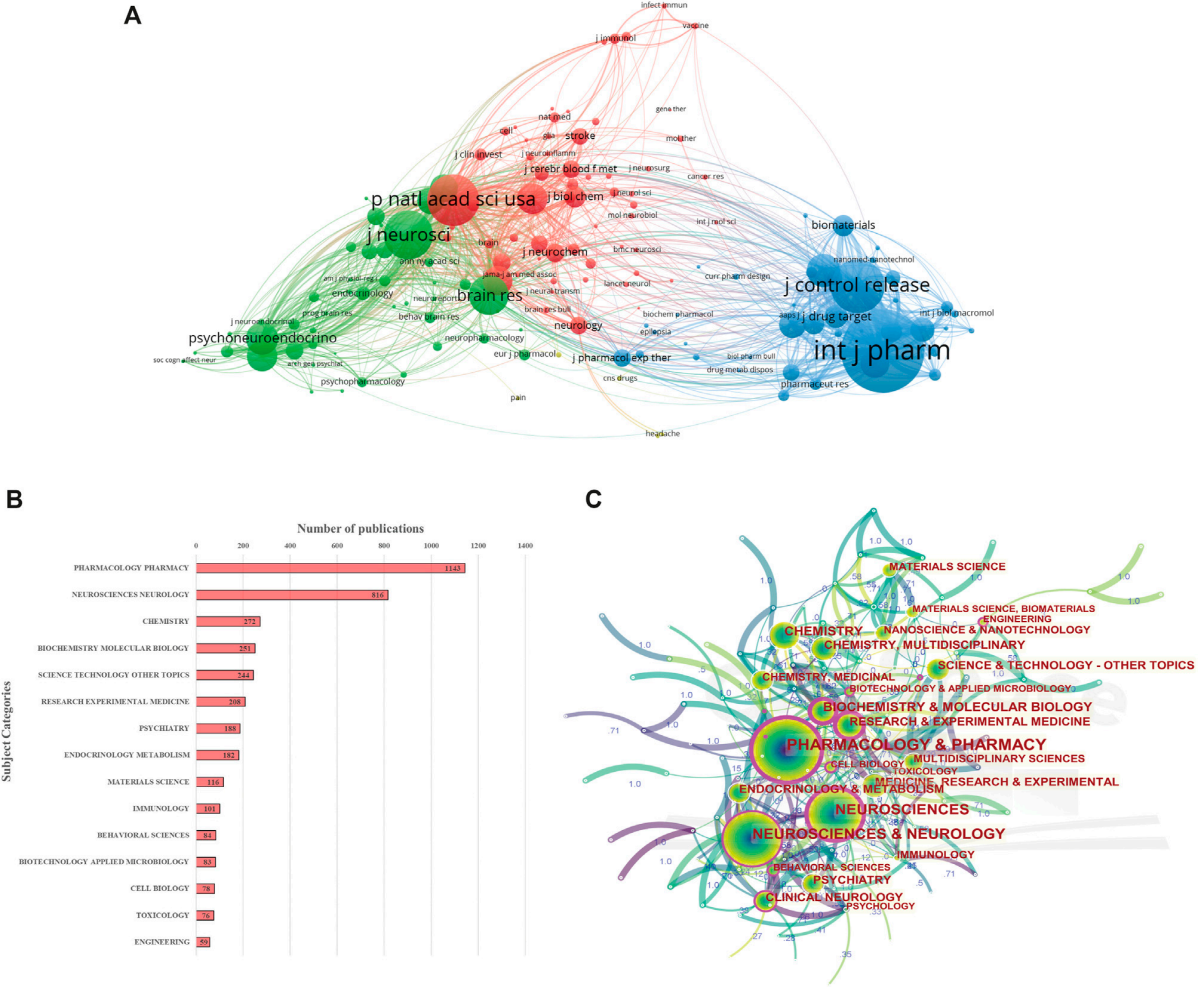
**(A)** Mapping on the 154 identified journals by co-citation analysis using VOS viewer. **(B)** The top 15 subject categories covered by all the included journals, and the quantity of publications for each subject category. **(C)** Co-occurring subject categories network of intranasal delivery research using CiteSpace.

Compared with the quantity of publications, the citation frequency is usually considered to be a better measure to evaluate a journal’s influence. Therefore, the co-citation network among journals was further analyzed using VOS viewer, and produced a visualization map of the co-cited journals, as indicated in Figure 7A. Only journals with a minimum of 200 citations were visualized. The network map contained 154 nodes, 11,704 links and 4 clusters. The top five journals with largest TLS were listed as follows: *International Journal of Pharmaceutics* (3,967.33), *Proceedings of the National Academy of Sciences of the United States of America* (2,774.88), *Journal of Controlled Release* (2,756.50), *Journal of Neuroscience* (2,577.14) and *Brain Research* (2,168.45). In summary, after careful consideration of the number of publications, citation frequency, and impact factor, the *International Journal of Pharmaceutics* is the most popular and influential journal in this domain.

Additionally, as shown in Supplementary Figure S2, we also create a dual-map overlay of the journals on intranasal administration research. In the dual-map overlays, the base map was built on the basis of 10,000 journals and the citing trajectories were generated after the input data (Chen and Leydesdroff, 2014b; Wu et al., 2021b). As described in previous study, the citing trajectories generated in the dual-map overlay module can describe the citation patterns and knowledge flows at the disciplinary level across almost the entire range of academic publications for a specific area (Wang et al., 2018). There were four core citation paths shown in Supplementary Figure S2, including one purple path and three orange paths. The one purple path indicates, articles published in Physics/Materials/Chemistry journals cited journals mainly in the field of Molecular/Biology/Genetics. The other three orange paths indicate, articles published in Molecular/Biology/Immunology journals cited journals mainly in the fields of Chemistry/Materials/Physics, Molecular/Biology/Genetics, and Psychology/Education/Social.

#### Subject Category Analysis

Each publication indexed in the WoS is assigned to one or multiple journal-based subject categories. Analysis of subject categories allows scholars to get an intuitive understanding of the main subjects involved in a research field (Chen et al., 2014a; Peng et al., 2020). In our dataset, a total of 81 unique subject categories were found. The most represented categories based on the quantity of publications were (Figure 7B): Pharmacology and Pharmacy (1,143 publications), Neurosciences and Neurology (816 publications), Chemistry (272 publications), Biochemistry and Molecular Biology (251 publications), and Science and Technology, Other Topics (244 publications). Figure 7C shows a visualization map of co-occurring subject categories. A purple ring on the edge of the node represents that this node has a high betweenness centrality, in other words, indicates its critical role of bridging the nodes it links. The top five subject categories ranked by centrality were Pharmacology and Pharmacy (0.29), Neurosciences (0.28), Research and Experimental Medicine (0.22), Radiology, Nuclear Medicine and Medical Imaging (0.22), and Biochemistry and Molecular Biology (0.21). Taking the above results in consideration, it can be noted that intranasal delivery research involves multiple disciplines. Pharmacology/Pharmacy and Neurosciences/Neurology were the hottest research categories in this field.

### Research Topics and Clusters of Global Publications

#### Analysis of Highly Cited References

Citation counts is a commonly used evaluation metric of scientometric analysis to quantify the relative impact of scientific papers in a subject area, as the influence and recognition of a paper is usually proportional to number of citations that the paper received (Li et al., 2021; Wu et al., 2021a). It is often assumed that highly-cited publication refers to high-quality studies with great influence and innovation in a certain field, which is also regarded as the knowledge base and essential readings for scholars wishing to engage in this field. Table 4 shows the details of the top 10 high-cited original articles in the research scope of intranasal administration. These studies were all published between 2004 and 2015, and seven were published prior to 2010. The majority of the articles were published in Neuroscience and Neurology journals such as *Journal of Neuroscience*, *Archives of Neurology*, *Neuron*, and *Neurology*. Of these, two papers were cited over 1,000 times with all the top 10 cited 400 times or more.

**TABLE 4:** Top 10 high-cited original articles in the research scope of intranasal administration.

Ranking	Title	Total citations	Journal	First author	Year
1	Oxytocin increases trust in humans	2,047	*Nature*	Kosfeld M.	2005
2	Oxytocin modulates neural circuitry for social cognition and fear in humans	1,075	*J. Neurosci*	Kirsch P.	2005
3	Oxytocin improves “mind-reading” in humans	849	*Biol. Psychiatry*	Domes G.	2007
4	Intranasal insulin therapy for alzheimer disease and amnestic mild cognitive impairment a pilot clinical trial	760	*Arch. Neurol*	Craft S.	2012
5	Oxytocin shapes the neural circuitry of trust and trust adaptation in humans	741	*Neuron*	Baumgartner T.	2008
6	Treatment of brain inflammatory diseases by delivering exosome encapsulated anti-inflammatory drugs from the nasal region to the brain	614	*Mol. Ther*	Zhuang X.	2011
7	Exosomes as drug delivery vehicles for Parkinson’s disease therapy	573	*J. Control Release*	Haney M. J.	2015
8	Intranasal insulin improves cognition and modulates beta-amyloid in early AD	539	*Neurology*	Reger M. A.	2008
9	Intranasal insulin improves memory in humans	479	*Psychoneuroendocrinology*	Benedict C.	2004
10	Effects of intranasal insulin on cognition in memory-impaired older adults: Modulation by APOE genotype	434	*Neurobiol. Aging*	Reger M. A.	2006

The work by Kosfeld et al. (2005), that explored the CNS effects of intranasal administration of oxytocin in humans, had the largest number of citations (2,047 times). The study confirmed that a single dose of 24 IU intranasal oxytocin could cause a significant increase in trust among humans compared with placebo group, and thereby greatly increase the benefits from social interactions during a trust game. To be more specific, 45% of the participants in the oxytocin group showed the maximal trust level, whereas only 21% in the placebo group, suggesting oxytocin played a vital role in the biological basis of prosocial approach behavior (Kosfeld et al., 2005). Additionally, the second, third, and fifth ranked articles (Kirsch et al., 2005; Domes et al., 2007; Baumgartner et al., 2008) further studied the role of intranasal oxytocin in social cognition and behavior. In particular, it was found that function of human amygdala was strongly modulated by oxytocin, which could potently reduce activation of the amygdala, the midbrain regions, and the dorsal striatum, and also reduce the coupling of amygdala to brainstem regions confirmed by using functional magnetic resonance imaging (Kirsch et al., 2005; Baumgartner et al., 2008). These findings indicated a neural mechanism of oxytocin in mental disorders such as social phobia or autism, and quickly triggered a series of subsequent studies.

Apart from the aforementioned highly cited studies of intranasal oxytocin, several studies on intranasal insulin have also received significant attention from many scholars (Benedict et al., 2004; Reger et al., 2005; Reger et al., 2008; Craft et al., 2012). Mounting evidence suggests that disturbances in insulin signaling pathways in the brain promote the development and progression of some neurodegenerative diseases, such as Alzheimer’s disease (AD), driving researchers to target this circuitry by means of intranasal insulin. Benedict et al. (2004) carried out a double blind clinical trial, in which the healthy subjects were randomly assigned to 8 weeks of intranasal insulin or placebo. The study found that prolonged intranasal administration of insulin could improve memory and mood (such as reduced anger and enhanced self-confidence) in the absence of systemic side effects. Subsequently, Craft et al. conducted a randomized, double-blind, placebo-controlled trial to examine the effect of intranasal insulin administration on adults with amnestic mild cognitive impairment (MCI) or AD. They found that the administration of intranasal insulin could stabilize or improve the cognition function and preserve the cerebral metabolic rate of glucose in regions affected by AD (Craft et al., 2012). Similar conclusions were reached in two other clinical studies conducted by Reger and others (Reger et al., 2005; Reger et al., 2008). Moreover, their results also showed that intranasal insulin administration was able to improve the verbal memory of AD participants without changes in plasma insulin or glucose levels.

The sixth and seventh ranked articles reported the findings on exosomes as drug vehicles for intranasal delivery (Zhuang et al., 2011; Haney et al., 2015). Exosomes, comprised of natural lipid bilayers, are naturally occurring nanosized vesicles with a diameter less than 100 nm (Bunggulawa et al., 2018). Noteworthy, exosomes possess unique properties, such as excellent biological acceptance and the ability to cross biological barriers (Lai and Breakefield, 2012; Bunggulawa et al., 2018). Therefore, exosomes have been exploited as drug vehicles in several investigations. For example, Zhuang et al. (2011) reported that exosomes encapsulated anti-inflammatory low molecular agent, curcumin, were proved to protect mice from brain inflammation induced by lipopolysaccharides. Another highly cited study published by Haney et al. (2015) also developed a novel exosomal-based delivery system for a potent antioxidant, catalase, to treat Parkinson’s disease (PD). And a considerable amount of this catalase-loaded exosomes were detected in PD mouse brain after intranasal administration, in turn, exert significant neuroprotective effects.

#### Analysis of References With Citation Burst

Although the top 10 most cited articles above were served as landmark articles due to the significance of their contributions, generally speaking, there was a modest correlation between the number of years since publication and the number of citations, that is, the earlier an article is published, the more citations it may have (Zyoud et al., 2016; Ma et al., 2020). As a consequence, some recently published high-quality studies cannot be identified solely by citation counts. In order to track and capture the evolution of research hotspots, the analysis of documents burst detection was conducted through CiteSpace. In general, articles with citation bursts mean that they have received special attention from associated academic circles in a past period (Wu et al., 2021a). Supplementary Figure S3 illustrates the top 25 references with the strongest citation bursts. The minimum duration of the burst was set for five years, and a red line segment represents the initial and final year of the burst duration.

References with citation bursts first appeared in 2005 due to the publications in 2004, and continued through 5 years (Benedict et al., 2004; Illum, 2004; Ross et al., 2004; Thorne et al., 2004). Of these, Thorne et al. (2004) investigated the possible pathways and mechanisms for the transportation of insulin-like growth factor-I (IGF-I) from the nasal cavity to the CNS. Their findings suggest that intranasal administration of IGF-I was able to bypass the BBB via olfactory- and trigeminal-associated extracellular pathways to elicit biological effects in the brain. An elegant review by Illum, (2004) summarized the evidence that confirmed the existence of a direct pathway from nose to brain. Benedict et al. (2004), as described earlier, demonstrated that intranasal insulin could improve memory and mood in the healthy subjects. While Ross et al. (2004) found that intranasal administration of IFNβ-1b to the brain provided a non-invasive method for the treatment of multiple sclerosis. More remarkably, three review articles have identified as the strongest burst keyword since 2015, and the burst remains ongoing (Djupesland et al., 2014; Kozlovskaya et al., 2014; Mittal et al., 2014). Integrated reviews can assist researchers to acquire research fronts and keep the abreast of knowledge-based developments. A large amount of citation bursts of reviews suggest that the importance of review articles has increasingly been recognized by the academic community.

#### Analysis of Co-Occurrence Keywords

**FIGURE 8:**
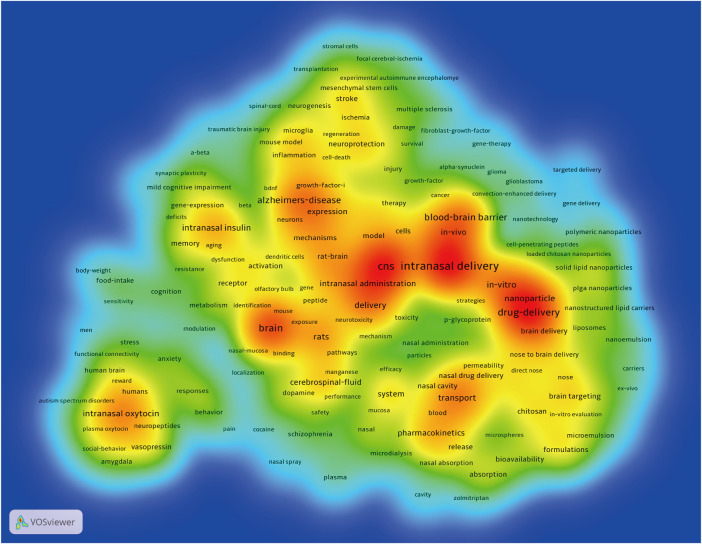
A density visualization map of included keywords using VOS viewer. The depth of the color was positively correlated with the occurrences of keywords.

Keywords are usually standardized representative terms selected to express the subject matter of a paper, and keyword co-occurrence analysis is a common scientometric method to highlight the most important research keywords that can represent the core content and provide a reasonable description of knowledge structures and thematic evolution of a certain field (Deng et al., 2020). The keyword co-occurrence network map was constructed and visualized with VOS viewer software. A total of 11,812 keywords were extracted from the 2,928 articles after merging some synonym keywords and filtering out keywords with general meaning manually. Only keywords with a minimum of 20 co-occurrences were visualized. Of the 247 keywords that met this threshold in a density visualization map (Figure 8 and Supplementary Table S3), the most frequently used keywords were “intranasal delivery,” “CNS,” “brain,” “drug-delivery,” “blood-brain barrier,” “Alzheimer’s-disease,” and “nanoparticle,” and so on. Of note, many of these keywords have already been presented in references with highly cited or citation burst.

**FIGURE 9:**
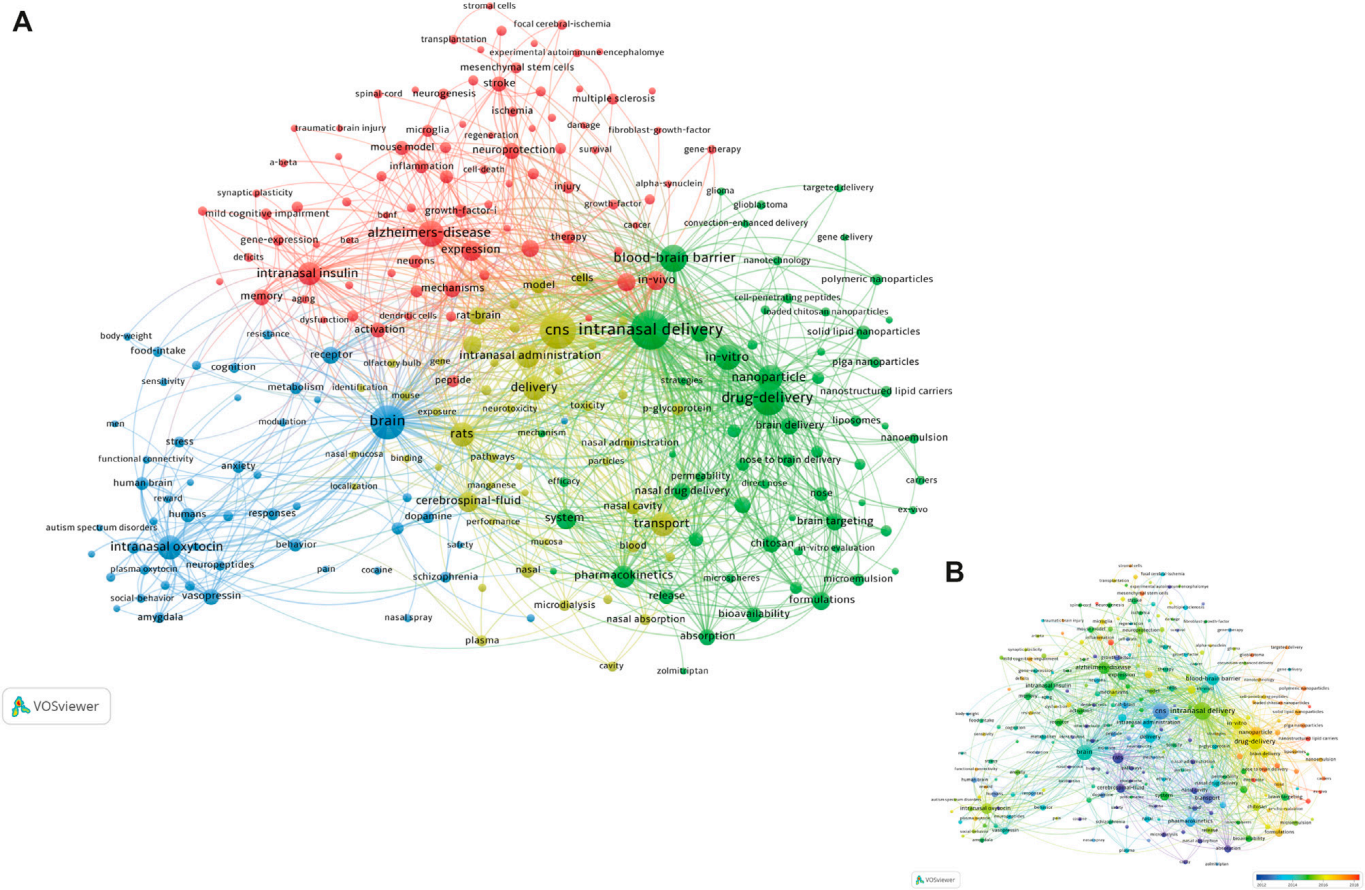
**(A)** Network visualization map of keyword co-occurrence analysis using VOS viewer. All keywords are labeled. The size of the node reflects the occurrence frequency of a certain keyword. The larger the size of the node is, the more frequently the keyword co-occurs. VOS viewer marks keywords with different colors, and the color of the nodes and labels indicates the cluster in which they belong to. Closely related keywords are grouped into one cluster with the same color. The higher the quantity of co-occurrences of two keywords, the closer will they be located in the network. **(B)** Overlay visualization map of keyword co-occurrence analysis. The meanings of the node and label in this map are the same as in Figure 10A. However, the color of each node in this map indicates the AAY of the keyword in the article according to the color gradient in the lower right corner.

Additionally, the clustering function in VOS viewer is able to divide the whole co-occurrence network into several clusters, and find the focus of recent research. Keywords with higher correlations are more likely to be put into the same cluster with the same color (Deng et al., 2020; Wu et al., 2021a). As shown in the network visualization map (Figure 9A), all these selected keywords could be roughly divided into four main topics: cluster 1 (red nodes, upper side of the network); cluster 2 (green nodes, bottom right of the network); cluster 3 (blue nodes, bottom left of the network); cluster 4 (yellow nodes, bottom middle of the network).

#### Cluster 1: Intranasal Drug Delivery for Neurodegenerative and Other Neurological Disorders

The red cluster (cluster 1) was the largest cluster with 85 keywords, and the primary keywords were Alzheimer’s-disease, intranasal insulin, Parkinson’s-disease, *in vivo*, neuroprotection, stroke, memory, oxidative stress, neuroinflammation, therapy, mouse model, and growth factor I (Rehman et al., 2019; Rhea et al., 2020; Su et al., 2020). Based on the above results, we could summarize that this cluster mainly has publications that focus on intranasal drug delivery for treatment of neurodegenerative and other neurological disorders (Benedict et al., 2004; Reger et al., 2005; Lochhead and Thorne, 2012). Of particular note is the keywords “Alzheimer’s-disease” and “intranasal insulin” have the highest co-occurrence frequencies, suggesting that intranasal insulin as a treatment for AD has attracted a lot of attention from researchers. And this result is consistent with the analysis of highly cited references (Reger et al., 2008; Craft et al., 2012).

#### Cluster 2: The Study of Nasal Drug Delivery Systems

Cluster 2 was the second largest cluster with 64 keywords. This cluster mainly focused on the study of nasal drug delivery systems including the following keywords: intranasal delivery, drug delivery, blood brain barrier, nanoparticle, *in vitro*, pharmacokinetics, system, formulations, chitosan, release, absorption, solid lipid nanoparticles. Despite numerous interesting features of intranasal delivery, as stated above, there are also several obvious drawbacks to this administration route, such as short drug residence time due to the existence of mucociliary clearance mechanisms, the limited drug permeability in nasal mucosa, and limited capacity of the nasal cavity (Costa et al., 2021; Froelich et al., 2021). In order to improve the effectiveness of intranasal drug delivery, different drug carriers and excipients including mucoadhesive formulations (Seju et al., 2011), lipid nanoparticles (Battaglia et al., 2018), nanostructured lipid carriers (Agbo et al., 2021), nanoemulsions (Costa et al., 2021), micelles (Wang et al., 2020) and microemulsions (Froelich et al., 2021) have been employed to enhance drug absorption and permeability, and protect drug from enzymatic degradation. Whereas most of these methods are currently under investigation in preclinical or early clinical stages, and successful reports to date have been confined to animal models (Cassano et al., 2021). Clearly, more detailed studies, particularly *in vivo* studies, are needed to better evaluate their efficacy and safety profile in the future.

#### Cluster 3: Intranasal Drug Delivery for Neuropsychiatric Disorders

Cluster 3 contained 50 keywords, mainly involved in intranasal delivery studies on neuropsychiatric disorders. The primary keywords were brain, intranasal oxytocin, vasopressin, receptor, schizophrenia, anxiety, stress, and behavior. Social-emotional functioning abnormalities are important transdiagnostic features and core symptoms of several neuropsychiatric disorders such as anxiety, depression, schizophrenia, and autism spectrum disorder. Mounting evidence suggests that anomalies in social-emotional processing in neuropsychiatric disorders may share underlying mechanisms, such as the oxytocin system (Davies et al., 2016; Katare et al., 2017). Numerous preclinical studies have found that intranasal oxytocin could improve social and emotional functioning by acting on central and medial amygdala (Kirsch et al., 2005; Domes et al., 2007; Baumgartner et al., 2008). However, up to date, evidence regarding the potential utility of intranasal oxytocin in treating several psychiatric disorders, including schizophrenia, anxiety and depression is still inconsistent (De-Cagna et al., 2019; Zheng et al., 2019). Further randomized controlled trials with larger samples and/or long-term administration of intranasal oxytocin are required to better elucidate its potential efficacy.

#### Cluster 4: Pathways and Mechanisms of Intranasal Delivery

Cluster 4 was the smallest cluster with 48 keywords regarding the pathways and mechanisms of intranasal delivery. The main keywords in this cluster were: CNS, delivery, transport, cerebrospinal fluid, intranasal administration, rats, nasal cavity, olfactory pathway, and p-glycoprotein. Numerous studies have investigated the potential mechanisms of direct nose to brain targeting (Patel et al., 2016; Crowe et al., 2018). Three major pathways are as follows: one is the systemic pathway by which some agents are absorbed into the bloodstream from the nasal cavity, and subsequently reaches the CNS by penetrating the BBB. The others are direct pathways in which drugs travel from the nasal cavity to cerebrospinal fluid and brain tissue through the olfactory nerve pathway and the trigeminal nerve pathway. Nevertheless, until now, the exact mechanism of this route is not fully known. Limited space prevents us from discussing the underlying pathways and mechanisms of drugs from nasal cavity to brain in detail. Many excellent reviews about this part are available for interested readers (Lochhead and Thorne, 2012; Crowe et al., 2018; Erdő et al., 2018; Agrawal et al., 2020).

#### Analysis of Changes in Research Hotspots

Figure 9B **(**a clearer image was provided in Supplementary Figure S4 **)** illustrates the overlay visualization map of keyword co-occurrence analysis. The meanings of the node and label in this map are the same as in the network visualization map. However, the color of each node in this map indicates the AAY of the keyword in the article (Wu et al., 2021a). According to the color gradient in the lower right corner, the nodes coded with dark blue represents the keywords appeared relatively earlier upon time course before or around 2012, whereas keywords that appeared around 2016 were coded with yellow color and those frequently used around or after 2018 appeared in red. Combining the specific location of four clusters from Figure 9A, it can be seen that, early research around the year 2012, “pathways and mechanisms of intranasal delivery (cluster 4)” had drawn much attention among investigators of this field. Afterward, “intranasal drug delivery for neurodegenerative, neuropsychiatric, and other neurological disorders (cluster 1 and cluster 3)” have progressively gained importance around 2015, and some areas remain the hotpots until today, for example, “neuroinflammation (AAY = 2017.90) (Rhea et al., 2020),” “mesenchymal stem cell (AAY = 2017.62)” (Aguilera et al., 2021; Vaes et al., 2021), and “mild cognitive impairment (AAY = 2016.85) (Craft et al., 2012).” Also, it needs to be noted that keywords in “cluster 2: the study of nasal drug delivery systems” had the largest AAY compared with other clusters, and the keywords of “nanocarriers (AAY = 2018.22) (Rehman et al., 2019),” “nanostructured lipid carriers (AAY = 2018.11) (Agbo et al., 2021),” “loaded chitosan nanoparticles (AAY = 2017.52) (Zhao et al., 2017),” and “PLGA nanoparticles (2017.41) (Nanaki et al., 2020)” were mainly found in the early years. These results indicate that the research focus of this field has shifted from “cluster 4: pathways and mechanisms of intranasal delivery” to “cluster 2: the study of nasal drug delivery systems, especially the nanostructured and nano-sized carrier systems” (Ali et al., 2010; Battaglia et al., 2018; Pires et al., 2020).

### Emerging New Hotspots and Frontiers

**FIGURE 10:**
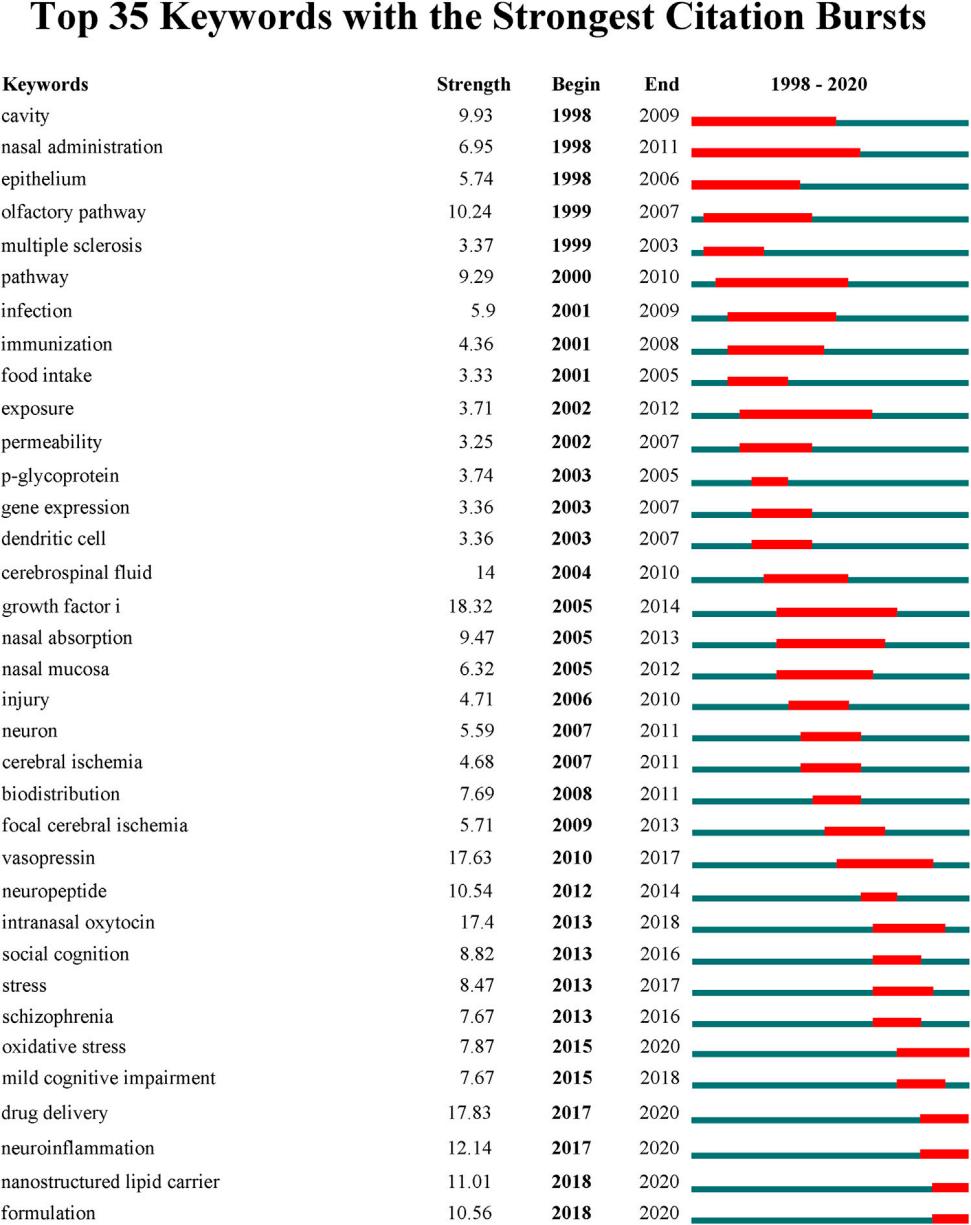
Top 25 keywords with the strongest citation bursts generated by Citespace.

Burst detection was also applied to detect keywords that had a surge of their appearance for a specific period of time. And it is usually considered to be another important indicator to determine the research hotspots, emerging trends and research frontiers over time (Chen et al., 2010). By using Citespace, we have analyzed the significant burst keywords with research implications between 1998 through 2020. A total of 35 keywords with citation burst were detected (Figure 10). Similar to the results described above, the evolution of the burst keywords during the past decade shows that the research focus has experienced a transition from the study of pathways and mechanisms of intranasal delivery to the study of intranasal drug delivery for CNS disorders and nasal drug delivery systems. On top of that, it is interesting to note that the burst of five keywords including “oxidative stress (2015–2020)” (Farfán et al., 2020; Katdare et al., 2020), “drug delivery (2017–2020) (Zhuang et al., 2011; Haney et al., 2015),” “neuroinflammation (2017–2020) (Rhea et al., 2020),” “nanostructured lipid carrier (2018–2020) (Agbo et al., 2021),” and “formulation (2018–2020) (Bors et al., 2020; Pires et al., 2020)” is still ongoing, suggesting that these research topics have gained great attention in recent years and also have great potential to continue to be the research focus and frontiers in the near future. Therefore, we can expect to see future work dissecting these topics, leading to more interesting scientific discoveries.

### Strengths and Limitations

This study provides a comprehensive overview of the knowledge framework, global trends and hotspots in the field of intranasal delivery research over the past 2 decades by using the scientific method of scientometric and visualized analysis. To the best of our knowledge, this is the first study to perform a scientometric analysis of peer-reviewed literature related to intranasal delivery research. We extensively and systematically searched the WoSCC database, and downloaded all relevant data within one day. The core journals, authors, countries/regions, funding agencies, institutions, and research topics and clusters were then identified to provide reference proposals for potential academic cooperation, funding-orientation guidance, and even scientific prizes in this field.

Nonetheless, there are still some inevitable limitations like other scientometric studies, that merit additional consideration in the interpretation of these results. First of all, we searched only WoSCC databases, which may omit some important studies from other large medical databases such as Scopus, PubMed and Embase. However, due to limitation of scientometric software, direct merging of two databases is difficult. Consequently, previous studies usually chose one of the databases as the main searching database, and of these, WoSCC is the most commonly used reference database for scientometric studies (Merigó and Yang, 2017; Li et al., 2021; Wu et al., 2021a; Wu et al., 2021b). Second, as this is a rapidly developing area of research, the importance of the contribution of recently published studies might have been underestimated due to their low citation frequency, although some of the latest high-quality studies were published in top grade journals. Finally, data generated from articles published in the current year (2021) was not included in our scientometric analysis as the database is continually updated and the dataset for this year is incomplete.

## Conclusion

Overall, intranasal administration route, as one of non-invasive and practical routes of drug delivery systems, that bypasses the BBB, provides an appealing approach alternative to traditional administration routes. In this study, the overall knowledge framework and current state of intranasal delivery research were visualized for the first time using a scientometric method. A total of 2,928 related documents including 2,456 original articles and 472 reviews were retrieved. The results clearly found that there has been a surge of interest in this field during the past decades. The United States dominated the field, reflecting in the largest amount of publications (971), the highest H-index (99), and extensive international collaborations. There was a very highly significant positive correlation between the quantity of publications and Gross Domestic Product (GDP) of countries (*r* = 0.951), indicating that much of the variation in the number of publications among different countries could be explained by economic factors. Jamia Hamdard contributed to most publications. Frey WH and Illum L were key researchers with the highest number of publications and citations, respectively. The *International Journal of Pharmaceutics* was the most influential academic journal, and Pharmacology/Pharmacy and Neurosciences/Neurology were the hottest research categories in this field. Based on keywords occurrence analysis, all these selected keywords could be roughly divided into four main topics: cluster 1 (intranasal drug delivery for neurodegenerative and other neurological disorders); cluster 2 (the study of nasal drug delivery systems); cluster 3 (intranasal drug delivery for neuropsychiatric disorders); cluster 4 (pathways and mechanisms of intranasal delivery). With respect to the analysis of AAY of the keywords, it can be concluded that the study of nasal drug delivery systems, especially the nanostructured and nano-sized carrier systems has attracted much attention in recent years. The keywords burst detection identified several keywords, including “oxidative stress,” “drug delivery,” “neuroinflammation,” “nanostructured lipid carrier,” and “formulation,” as new research hotspots, which have currently ongoing bursts and also have great potential to continue to be the research frontiers in the next few years. In summary, this study offers a comprehensive scientometric analysis of intranasal delivery research from a global perspective, and can serve as a starting point in drawing the attention of scholars and policymakers worldwide to further identify and contribute to the increasing scientific work in this domain.

## Data Availability

The original contributions presented in the study are included in the article/Supplementary Material, further inquiries can be directed to the corresponding authors.
