# Supplementary material for: Current State and Future Directions of Intranasal Delivery Route for Central Nervous System Disorders: A Scientometric and Visualization Analysis
Source: Front Pharmacol. 2021 Jul 12;12:717192. doi: 10.3389/fphar.2021.717192 (PMC8311521; doi:10.3389/fphar.2021.717192)
Supplement: Supplementary file 1 [file DataSheet1.docx]

Supplementary Material

# Supplementary Figures and Tables

## Supplementary Figure


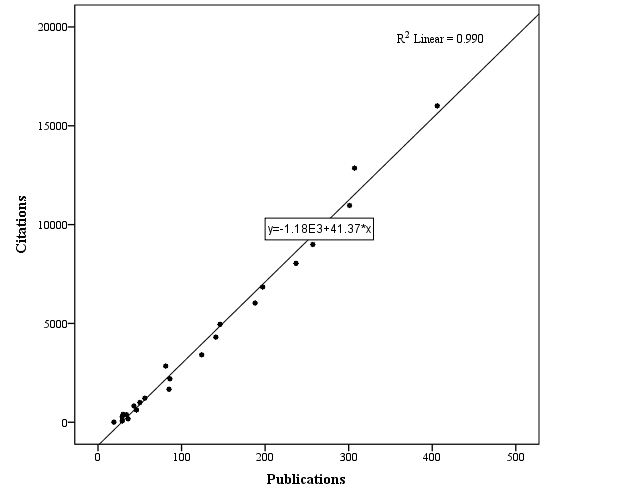


**Supplementary Figure 1.** Correlation analysis between publications and citations. The Correlation analysis was performed using Pearson’s correlation test.


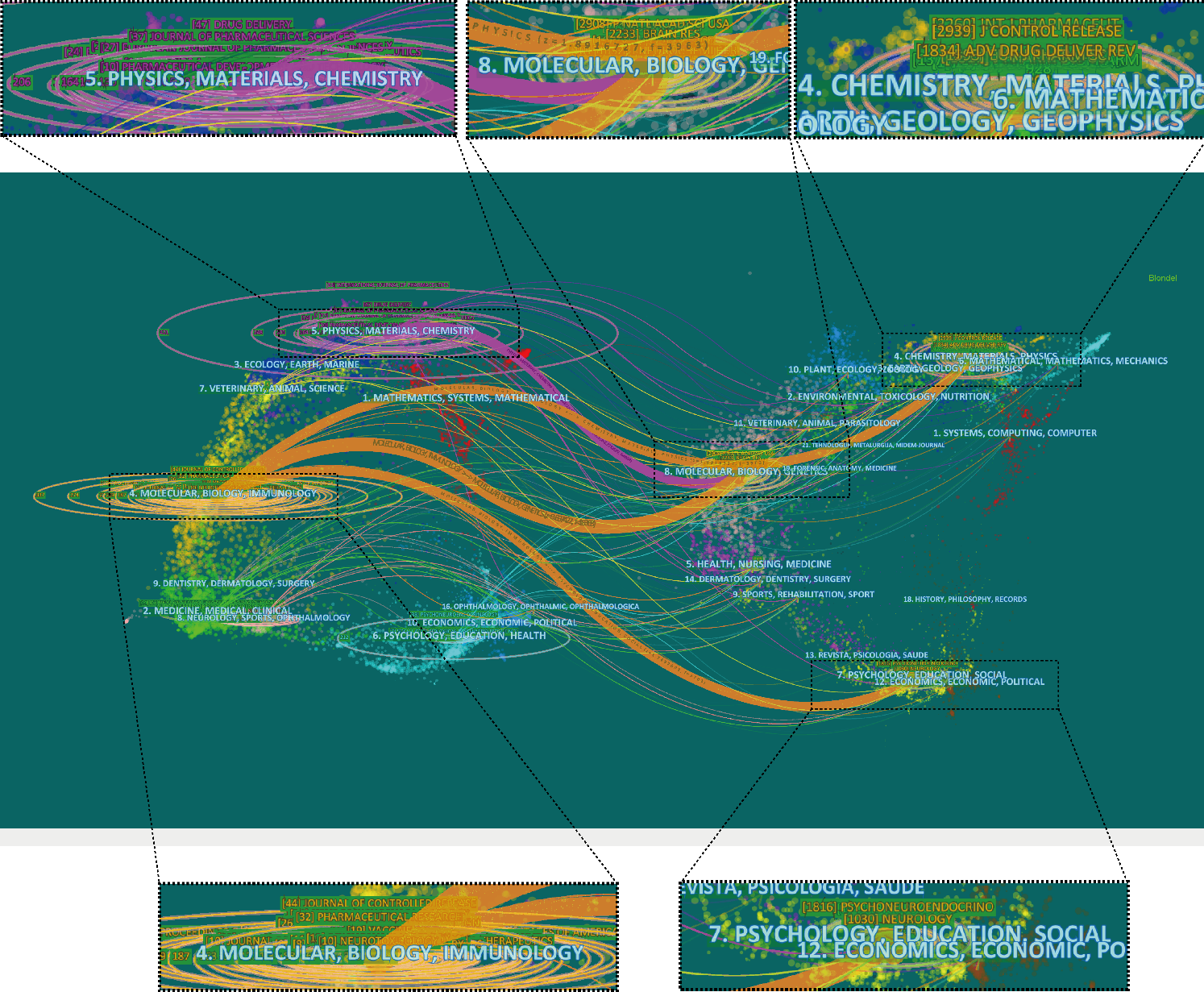


**Supplementary Figure 2.** The dual-map overlay of journals publishing intranasal delivery research generated by CiteSpace. The left and right side represent the citing journals and the cited journals, respectively. The line provides a function of citation connections, beginning from the citing journals and terminating at the cited journals. One can refer to (Chen et al., 2014b) for more explanation.


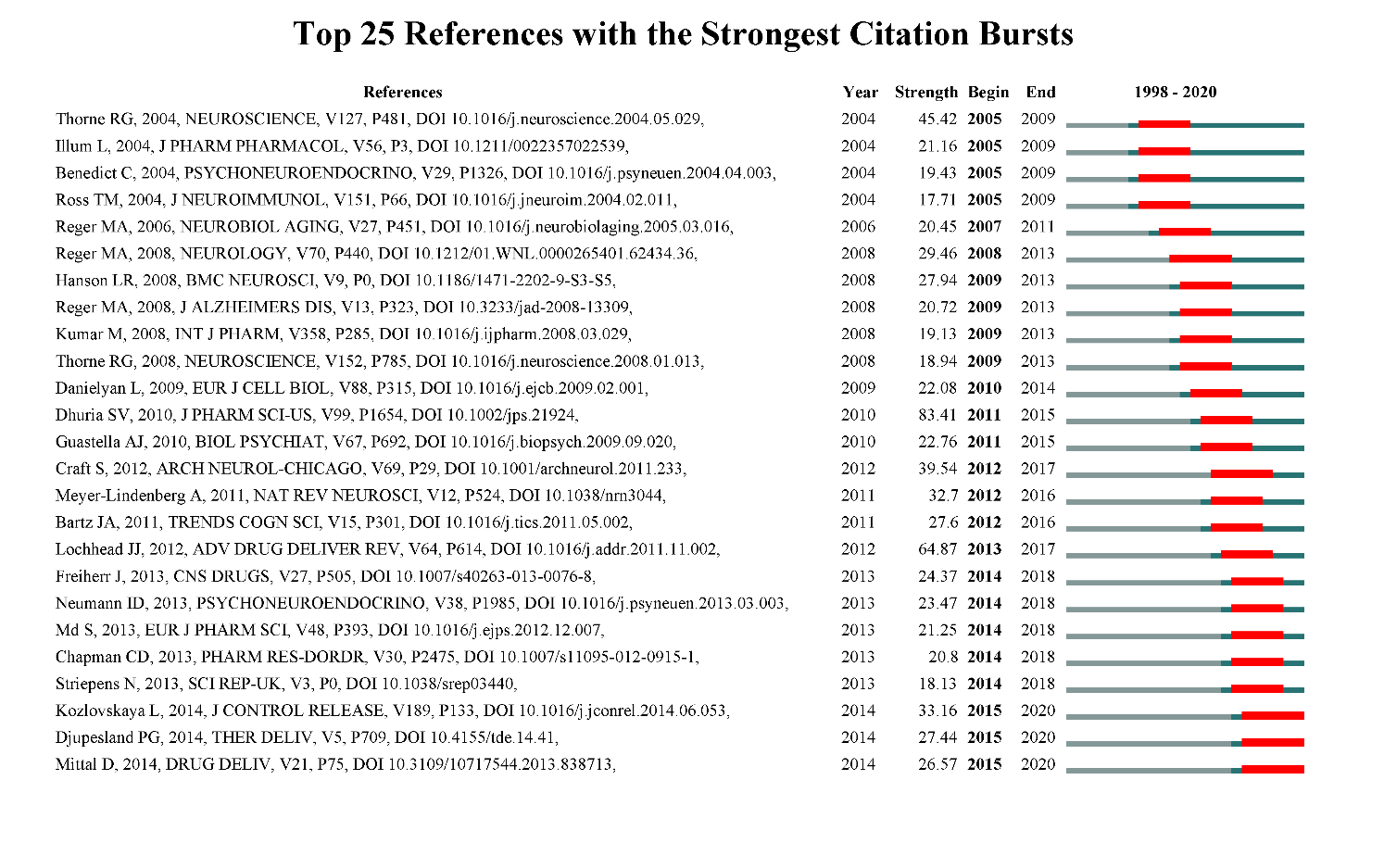


**Supplementary Figure 3.** Top 25 references with the strongest citation bursts generated by Citespace.


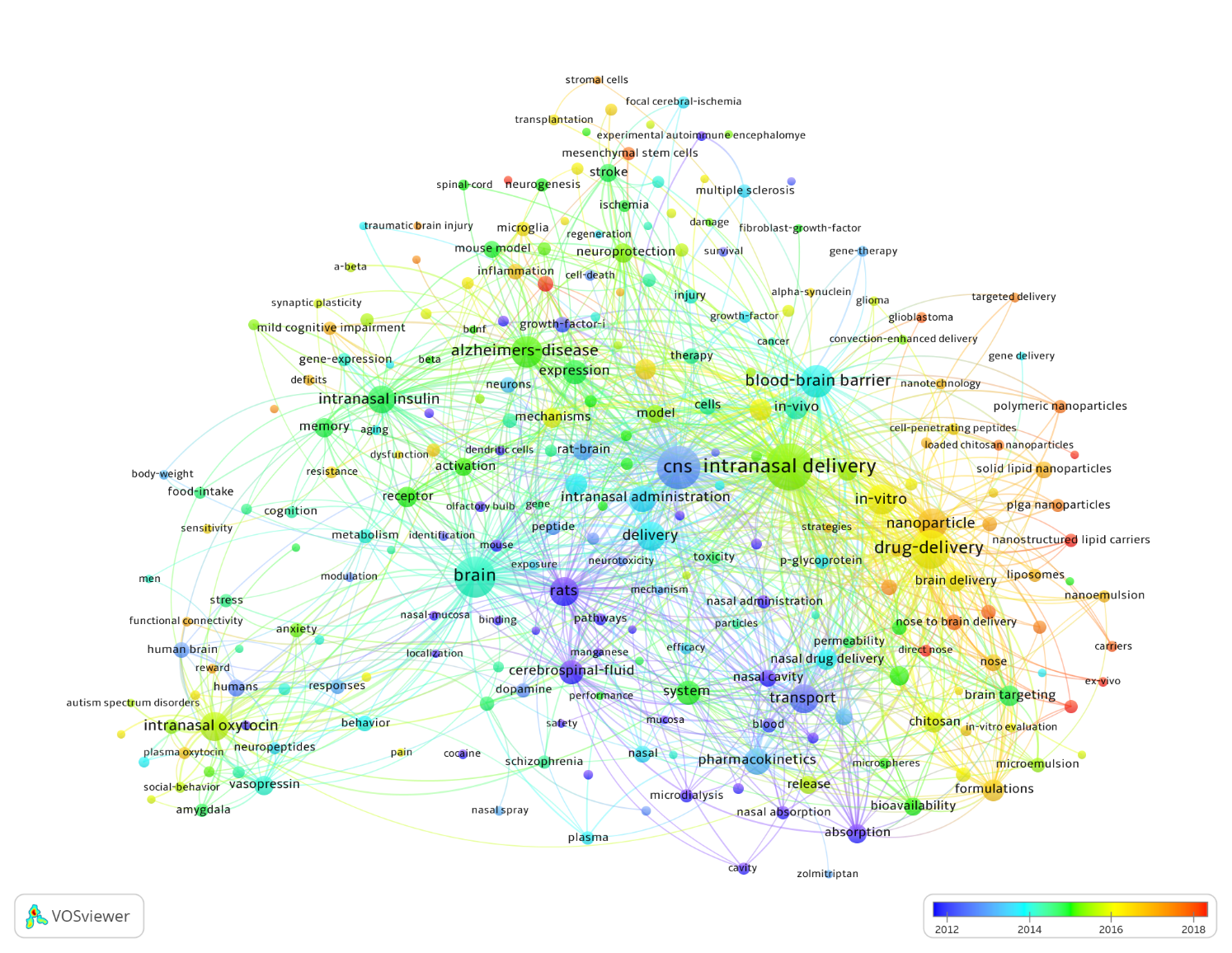


**Supplementary Figure 4.** Overlay visualization map of the keywords co-occurrence analysis generated by VOS viewer.

## Supplementary Table

**Supplementary Table 1.** Correlation analysis between the total number of publications, the economic and demographic indices of the countries.

|  | Total population | GDP |
| --- | --- | --- |
| Total number of publications | *r* = 0.486 | *r* = 0.951 |
|  | R^2^ = 0.236 | R^2^ = 0.904 |
|  | *P* = 0.030 | *P* = 0.000 |

GDP: Gross Domestic Product. Statistically significant (0.00 < *r* < 0.25: little if any correlation; 0.26 < *r* < 0.49: low correlation; 0.50 < *r* < 0.69: moderate correlation; 0.70 < *r* < 0.89: high correlation; 0.90 < *r* < 1.00: very high correlation)

**Supplementary Table 2.** The top 20 keywords with the most occurrences

| Keywords | Occurrences | Keywords | Occurrences |
| --- | --- | --- | --- |
| intranasal delivery | 735 | transport | 260 |
| CNS | 629 | intranasal oxytocin | 256 |
| brain | 546 | intranasal insulin | 232 |
| drug-delivery | 496 | intranasal administration | 213 |
| blood-brain barrier | 347 | pharmacokinetics | 209 |
| Alzheimer’s-disease | 317 | cerebrospinal-fluid | 181 |
| nanoparticle | 297 | expression | 170 |
| in-vitro | 291 | in-vivo | 164 |
| delivery | 270 | Parkinson’s-disease | 150 |
| rats | 269 | formulations | 141 |
